# Alternative proteins in low- and middle-income countries (LMIC) face a questionable future: will technology negate Bennett’s law?

**DOI:** 10.1016/j.cdnut.2023.101994

**Published:** 2023-09-01

**Authors:** Adam Drewnowski

**Affiliations:** Center for Public Health Nutrition, University of Washington, Seattle, WA, USA

**Keywords:** Bennett’s law, alternative proteins, animal protein, nutrition transition, protein transition, FAOSTAT, World Bank

## Abstract

Rising incomes across low-and middle-income countries (LMIC) lead to a lower consumption of starchy staples and create a growing demand for high-quality animal protein, an observation referred to as Bennett’s law. This dietary shift from plant-sourced to animal-sourced proteins has also been referred to as the LMIC *protein transition*. At this time, there are rising concerns that current livestock production is highly resource intensive and may not meet the growing global demand for high-quality protein. Alternative plant-based proteins, derived from new technologies and often fortified with micronutrients, are intended to close the LMIC nutrient gap. However, data from LMIC suggest that the income-driven selection of animal proteins is aspirational and varies by stage of economic development. Food balance sheets from higher-income countries indicate that meat consumption peaks only at very high incomes. Will plant-based alternative proteins satisfy the growing LMIC demand for animal-sourced foods, thereby negating Bennett’s law? Current evidence suggests otherwise.

## Introduction

The term nutrition transition refers to those dietary changes in low- and middle-income countries (LMIC) that follow economic development [1]. Richer countries and more affluent consumers abandon root crops and cereals to seek out more varied and more nutrient-dense diets with more meat, eggs, milk and dairy products [[1], [2], [3], [4]]. Although the total percentage of energy from protein remains roughly constant, dietary choices turn to high-quality proteins of animal origin, a phenomenon referred to as the protein transition [5]. Viewed as a subset of the broader nutrition transition, the protein transition is also income-driven. However, the choice of specific animal protein can vary widely depending on geographic region, tradition, religion, or culture [5].

Two opposing protein transitions are currently taking place globally [5]. Across LMIC, plant-based diets are giving way to more meats, eggs, and dairy. This protein transition is actively promoted by international agencies [6] and by local governments [7], aiming to improve LMIC diet quality and population health. At the same time, high-income countries propose to replace meats, eggs, and dairy with plant-based foods, including foods formulated with a variety of alternative proteins. Conventionally derived from soy, pulses, beans and legumes, grains, and nuts [8], alternative proteins can also be manufactured from more unusual fungi, green algae, and red seaweed [8]. That protein transition is also actively promoted by international agencies and by local governments [9], aiming to improve diet quality and population health.

It is the current attempts by rich-country actors [6] to impose plant-based protein diets across LMIC that raise some concerns. Such efforts may run counter to the laws of economics and ignore local and territorial aspirations, preferences, and food cultures [10]. Such efforts may be doomed to failure, unless suitable societal and technological solutions are found.

What learnings from high-income countries can be applied to those LMIC that are currently in different stages of the protein transition? Laws of economics may help predict future food demand. Bennett’s law [11] is the name given to the observation that, as incomes rise, people eat fewer root crops, legumes, and cereals and diversify their diets to include more animal-sourced foods and especially meat. The proportion of energy from root crops, legumes, and cereals declines accordingly whereas the proportion of energy from meats, eggs, and dairy increases. Effectively, Bennett’s law predicts that plant-based proteins will be replaced by animal proteins as an inevitable consequence of economic growth [1]. More affluent consumers seek out calories that are more expensive and more nutrient rich [2].

Based on recent analyses of FAO food balance sheets [12], some high-income countries have already achieved peak meat consumption [13]. Their adopting more plant-forward diets is likely to benefit both personal and planetary health. By comparison, most LMIC are nowhere near peak meat consumption and may not attain it for another 50 y [9]. In the meantime, micronutrient deficiencies that are associated with the traditional plant-based LMIC diets are still prevalent [14]. Among missing priority micronutrients are iron, zinc, calcium, vitamin A, and vitamin D, all commonly found in animal-source foods. The growing LMIC demand for animal protein is a very effective way to address multiple micronutrient deficiencies and other nutrient needs. Global meat demand is projected to increase substantially over the next decade [9].

That the growing global demand for high-quality animal protein may outpace population growth is an area of concern. Meeting that demand through livestock production will be highly resource intensive, if not ecologically devastating. Alternative proteins derived from soy, pulses, beans and legumes, and other more exotic plant sources may help minimize the environmental impact of animal protein production and close the LMIC nutrient gap. Meat analogs, in particular, are reputed to offer comparable nutrition at lower environmental (if not monetary) cost. The present question is whether modern technologies, aided by modern marketing, will be sufficient to stem the nutrient-driven and aspirational demand for animal protein and so negate the basic tenets of Bennett’s law? This commentary focuses on nutrition economics but also considers the diversity of the food supply across LMIC.

## The real Bennett’s Law 1941

Bennett’s law [11] in its original form was actually more circumscribed than is commonly supposed. The 1941 article began with the realization that starchy potatoes and cereals were substantially cheaper per 1000 kcal as compared to other foodstuffs. The term “potatoes” meant primarily cassava but also included yams, sweet potatoes, and white potatoes. The cereals were wheat, rye, rice, barley, oats, corn, millet, and sorghum. The next observation was that lower-income families resorted to potatoes and cereals whereas families with rising incomes replaced them with more expensive foods [11]. In other words, high dependence on low-cost carbohydrate calories at the household level was an indicator of low household incomes [11]. It followed at the macro scale that the national ratios of cereal-potato calories to total food calories would be a rough indicator of relative per capita levels of national income. That became known as Bennett’s law [11].

Bennett’s second proposition was that the national ratio of dietary energy from starchy staples to total food calories was a crude indicator of diet quality at the population level. The argument was that if a nation’s diet was composed of >80% root crops and cereals, then it was not nutritionally adequate or balanced and was most likely lacking in essential nutrients [11]. This observation has been interpreted to mean that rising incomes drove the demand for more nutrient-dense foods, including high-quality animal protein from meat. Later researchers have claimed that Bennett’s law reflects the universal desire for high-quality protein, more dietary variety, and more refined sugar [15]. Bennett did not mention refined sugar.

Bennett [11] noted that exact data to confirm those ideas were not available at the time. Since then, multiple analyses confirming Bennett’s law have relied on FAOSTAT food balance sheets from the Food and Agriculture Organization of the United Nations (FAO) and on gross domestic product (GDP) per capita figures from the World Bank. The present analyses used FAOSTAT data [12] for selected commodities for years 2019–2020 for low income (GDP <$1000), lower middle income (GDP $1000–$4000), upper middle income (GDP $4000–$13,000) and high-income countries (GDP >$13,000) as defined by the World Bank [12,13]. Additional analyses used 2013 FAO data for energy from plant and animal proteins in calories/capita/day by country, merged with 2013 country GDP values from the World Bank [16].

These data have well-known limitations. FAOSTAT food balance sheets are for commodities that are available or are allocated directly for human consumption in a given country, excluding possible uses for animal feed or biofuels. Although food balance sheets are supposed to correct for imports and exports, that is not always the case. Since the food supply data are aggregated at country level, it is not possible to look for foods available to population subgroups. Neither is it possible to assess the extent to which a country’s food supply satisfies the population’s nutritional needs. The FAOSTAT data capture current production trends [17] and have been used to predict future food demand. They have also been used as proxies for food consumption. For example, the EAT Lancet report [8] relies entirely on FAO country-level food balance sheets that had been transposed into consumption data at the individual level.

## FAOSTAT food protein sources by country income groups

**FIGURE 1:**
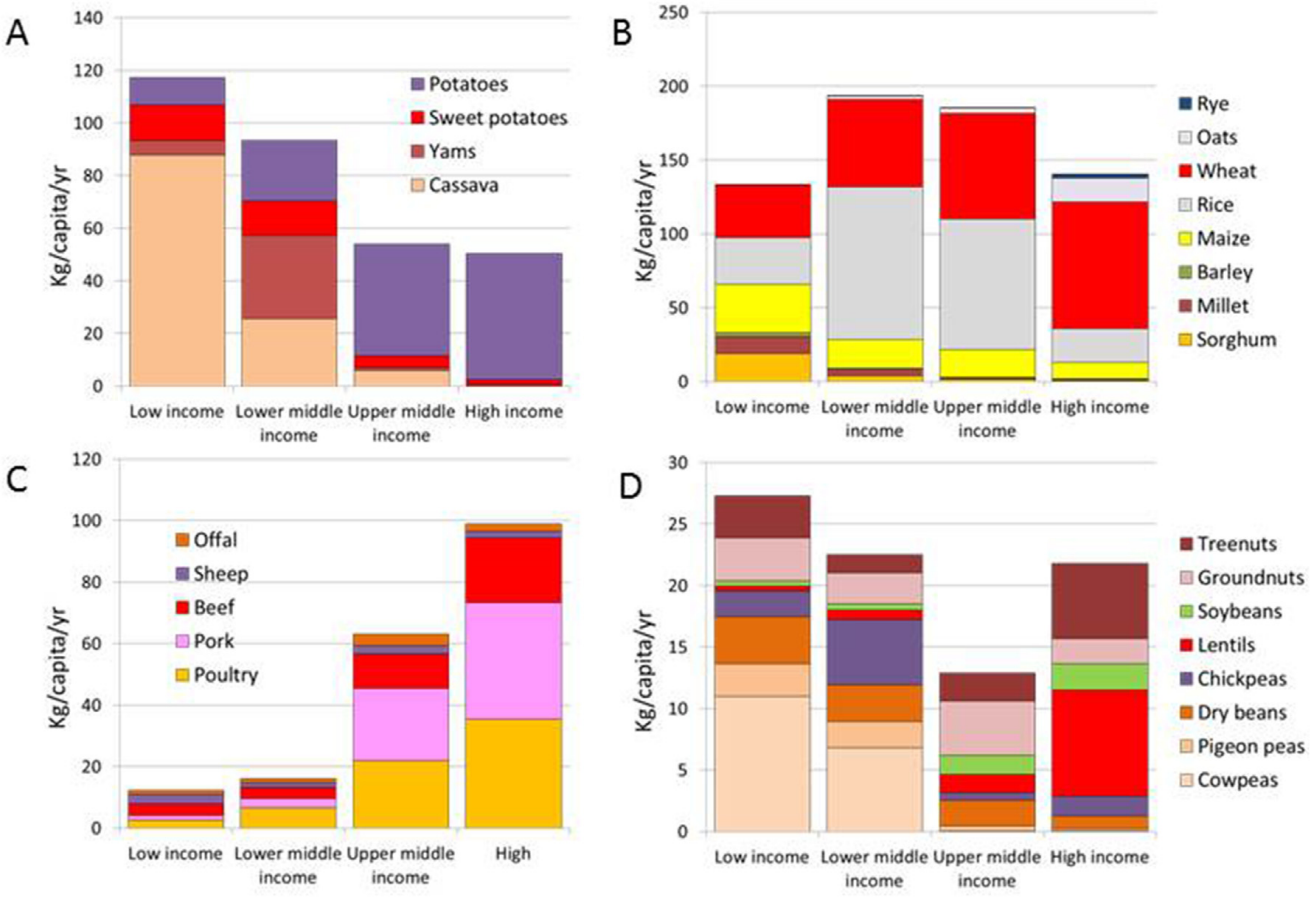
Roots and tubers (A), cereals (B), meats (C), and beans, pulses, legumes, and nuts (D) in kg/capita/year available for global human consumption. Data are FAOSTAT 2019–2020 by World Bank country categories by gross domestic product (GDP).

Figure 1 examines diverse commodities available for human consumption by country income category, as defined by the World Bank. The data are for 2019–2020 and are expressed in kg/capita/year. Bennett’s cereal-potato crops are shown in Figure 1A. Along with an overall decline, there is evidence for substitution within the root crops category. Whereas cassava, yams, and sweet potatoes predominate in low-income countries, yam production is higher in LMIC. By contrast, white potatoes are mostly associated with high-income countries. Some substitution is also occurring with grain crops. Figure 1B shows the income-driven shift from coarse grains (sorghum, millet, and maize) to rice, which had been noted in 1983 [18]. Rice is more prevalent in LMIC. In high-income countries, rice is replaced by wheat. Oats and rye are associated with high-income countries only. It is also worth noting that in the United States, wheat and corn are feed ingredients for the cattle, pork, and poultry industries.

These FAOSTAT food supply data are generally consistent with published reports of dietary intakes [17]. Before dietary energy from starchy staples begins its inevitable decline [18], there is evidence for substitution within the category. In Asia, the tropical root crops (cassava and sweet potato) along with coarse gains (maize, sorghum, and millet) have given way to rice as the primary food source [19]. Although some Southeast Asian countries still depend on rice, consumption patterns in richer Asian countries tend toward wheat. Rice production (and consumption) has declined in Southeast Asia. By contrast, rice production is growing in Africa where it is replacing cassava, a less desirable root crop.

Obviously, cassava and white potatoes have different areas of cultivation that are climate dependent. These agricultural production data confirm Bennett’s earlier findings [11] and seem to correspond to regional food consumption patterns.

Figure 1C provides food balance sheets for the type of meat by country income level. The biggest growth by far has been in the poultry sector (chicken) and pork. There has been an explosive growth in global poultry production, particularly in Africa, Asia, and Latin America since the 1990s. Bennett’s law does not specify a hierarchy of animal proteins.

Figure 1D shows FAOSTAT data for beans and peas, pulses (dry beans), groundnuts (peanuts), and tree nuts. Those are among the currently promoted alternative sources of plant protein. First, cowpeas, pigeon peas, and dry beans are associated with low-income countries. More chickpeas are consumed in LMICs; more groundnuts in upper middle-income, and more soy in upper middle- and high-income countries. There has been an enormous recent growth in high-income country production of lentils and tree nuts. The pulses are not destined for domestic consumption, but rather for export as potential sources of plant protein for LMIC [20].

The observed protein transition does not occur at the same rate across all LMIC, and consumption trends for animal protein vary by region. For example, higher incomes have not translated into higher fish consumption in Southeast Asia, where it is now associated with rural areas and lower education and incomes [21]. Instead, the regional trend seems to be toward mostly more chicken and dairy followed by pork and beef. Whereas beef is more prevalent in Japan, China, Korea, and Vietnam show a preference for pork. By contrast, trends in Africa may be toward poultry, eggs, and dairy.

Local supply issues can also play a role. When it comes to animal proteins from meat, poultry, and fish, there are problems with the perishable nature of the product, lack of processing facilities, and lack of cold storage. There are also social issues. The specific choice of animal protein is fraught with social and cultural meaning [6]. The choice of meat compared with eggs or dairy and the choice of beef as opposed to pork can depend on social conditions, tradition, and culture [21].

## FAOSTAT animal and plant proteins by country income group

**FIGURE 2:**
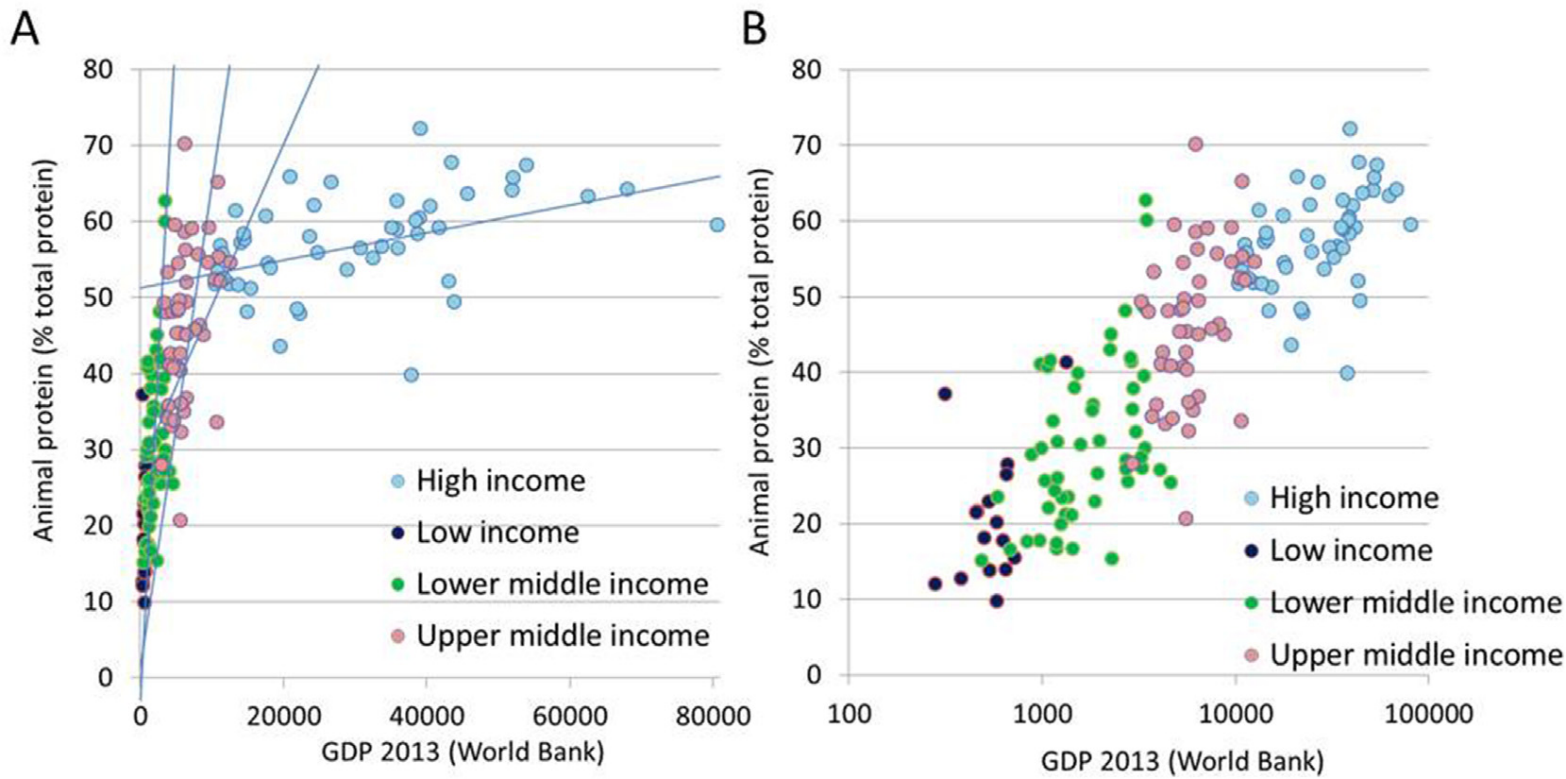
Percent animal protein from FAOSTAT and 2013 gross domestic product (GDP) values in US dollars from the World Bank by country. GDP data are plotted on linear (A) and logarithmic (B) scales.

The present analyses matched 2013 FAOSTAT data for 164 countries with World Bank incomes, also for 2013. GDP data are highly skewed and are conventionally presented following a log transformation. Both are presented in Figure 2A, B.

Figure 2A confirms the previously observed sharp increase in the share of energy from animal protein that is associated with rising GDP. However, the percentage of animal protein does not increase indefinitely, and there is a slowing down at highest GDP. At approximately $40,000 and above, percent of animal protein (as percentage of total) is stable, and higher incomes are no longer associated with higher ratios of animal to plant protein. Analogous data were taken as evidence that high-income countries have achieved peak meat consumption. However, only a handful of countries have 2013 GDP >$40,000. Those include Norway, Sweden, Denmark, Netherlands, Switzerland, Australia, Canada, and the United States. Those are also the countries that are now actively promoting plant-based proteins to the rest of the world.

## Alternative proteins in the LMIC context

The promotion of alternative proteins in place of meat, eggs, and dairy is most often justified with reference to health and the environment. The current consensus among high-income country actors is that healthy global diets ought to be largely plant-based with ample amounts of legumes, nuts, seeds, and whole grains and minimally processed vegetables and fruits. Meat is to be consumed sparingly or not at all, with additional limits placed on eggs and dairy [8]. Manufactured plant-based alternative proteins promise to provide high-quality protein at an affordable cost. Fortified with vitamins and minerals, plant-based burgers and plant-based dairy alternatives aim to satisfy the growing demand for desirable animal-source foods by providing equivalent nutritional value.

The larger promise is that alternative proteins, not all of them plant-based, may lower the environmental footprint of the global population diet. Here, the range of alternative proteins expands from beans and soy to include precision fermentation, cultured meat, and insect-derived proteins. Such products are designed to mimic those desirable proteins for which the demand is high and likely to increase.

Current attempts to stem the ongoing protein transition across LMIC need to be carefully evaluated. In many LMIC, undernutrition and hidden hunger remain major public health issues. The traditional plant-based diets in many regions of the world are associated with micronutrient deficiencies, notably in calcium, iron, zinc, vitamin A, iodine, and vitamins B2 and B12. Diets of starchy staples built around cassava, rice, and maize have been associated with lysine deficiency and amino acid imbalance. Some of these deficiencies can be remedied by the addition of small amounts of animal foods to the diet. It is not clear whether manufactured alternative proteins would be equally nutritious or equally acceptable.

Strict nutrition standards would need to be followed to integrate novel alternative proteins into dietary patterns. Products that are marketed as alternatives to meat, eggs, or dairy would need to be fortified to ensure that they are not nutritionally inferior to the original product. Animal proteins are typically associated with bioavailable iron, zinc, calcium, vitamins A (retinol) and B12, and other B vitamins. In high-income countries, plant-based alternatives to meat and dairy are often fortified with the relevant nutrients, but not always. Plant-based milks are fortified with calcium, vitamin A, and vitamin D. Plant-based meat alternatives are often fortified with iron, zinc, and vitamin B12, but not always. Protein quality and the bioavailability of calcium, iron, and zinc are issues of concern to LMIC and would need to be resolved.

## What is the future of alternative protein in low-income countries?

Local production of animal foods has been increasing in Africa and Asia, often with substantial support from the FAO. Studies agree that the income-driven demand for animal protein is rapidly increasing across LMIC and will continue to do so for the foreseeable future. As predicted by Bennett’s law, consumers purchase more nutrient-rich and more expensive calories as incomes rise. Their tastes run toward more chicken, eggs and dairy, pork, and in some cases, beef. These new diets provide an effective remedy for multiple micronutrient deficiencies, notably those in iron, calcium, zinc, and vitamins A and B12. LMIC populations may derive no immediate health benefits by replacing animal products with manufactured foods containing plant-based proteins.

By contrast, some high-income countries that over-consume protein and are already at peak meat consumption would probably benefit from a diet with more plant-based foods. The present analyses show that in a handful of countries with GDP above $40,000 there is a dissociation between incomes and percentage of animal-source protein. In those countries, plant proteins are about 30% of the total and plant-based meat analogs and plant-based milk alternatives appear to be a growing market segment.

Some high-income countries have also made major investments in the production of pulse crops that are intended for export to the LMIC rather than for domestic consumption. The question is whether the push for alternative plant-based proteins for global use will compete with local and regional agriculture. What is more, LMIC consumers may not aspire to consume alternative protein foods produced in rich countries and manufactured from peas, lentils, or soy.

In conclusion, it is likely that Bennett’s law will prevail, regardless of current efforts to reverse the global protein transition and replace desirable animal proteins with manufactured plant-based foods. It may take decades for LMIC to reach the peak meat consumption that is currently the privilege of some countries that are farther along the economic scale. However, the animal protein of choice across LMIC will not necessarily be the traditional beef. The current global trends are toward improved LMIC livestock production technologies and toward more chicken, more pork, and more dairy. Alternative proteins will need to compete with local agriculture and local cultures and food ways.

## Acknowledgments

I thank Klaus Kraemer, Kesso G. van Zutphen-Kuffer, Jacquelyn R. Bedsaul, and Jimena Monroy-Gomez from *Sight and Life* Foundation for their critical review of this manuscript and coordination of this supplement and Nitya Vissamsetti for contributing to reference formatting.

## Author contributions

The sole author was responsible for all aspects of this manuscript.

## Conflict of interest

AD is a member of Scientific Advisory Boards for Nestlé, BEL, FrieslandCampina Institute, and the National Pork Board and is an invited member of the Carbohydrate Quality Expert Panel supported by Potatoes USA. AD has received grants, contracts, and honoraria from entities both public and private, with an interest in dietary nutrient density and nutrient profiling of foods.

## Funding

The present analyses of open access FAO and World Bank datasets were supported by *Sight and Life* Foundation.

## Data availability

Data described in the manuscript, code book, and analytic code are publicly and freely available without restriction at FAOSTAT (https://www.fao.org/faostat/en/#data/FBS); Our World in Data (https://ourworldindata.org/grapher/share-of-calories-from-animal-protein-vs-gdp-per-capita) and the World Bank (https://data.worldbank.org/indicator/NY.GDP.PCAP.CD).
